# Mesenteric ischaemia ocurring as a late complication after-aorto-femoral bypass

**DOI:** 10.4103/0019-5049.76600

**Published:** 2011

**Authors:** Neeti Makhija, Raveen Singh, Usha Kiran, Madhava Kekani, Minati Choudhury

**Affiliations:** Department of Cardiac Anaesthesia, CN Centre, All India Institute of Medical Sciences, Ansari Nagar, New Delhi, India

**Keywords:** Atherosclerosis, coronary artery disease, mesenteric ischaemia, peripheral vascular disease

## Abstract

Patients with coexisting peripheral vascular disease and coronary artery disease constitute a high risk surgical group. Perioperative management of such patients is an anaesthetic challenge. A 57-year-old male presented with critical limb ischaemia and impending gangrene of the right lower limb. Associated coronary artery disease with triple vessel involvement was diagnosed on coronary angiography. This patient underwent an aorto-femoral bypass. The postoperative course was complicated by the development of mesenteric ischaemia requiring emergency laparotomy and bowel resection.

## INTRODUCTION

Perioperative management of patients with peripheral vascular disease is challenging. Extensive atherosclerotic involvement of the vascular system including coronary, renal, mesenteric and cerebral vessels predisposes it to increased perioperative mortality and morbidity in the form of myocardial infarction, congestive heart failure and renal failure.[1] We report a case of aortoiliac disease presenting as impending foot gangrene in a patient who underwent an aortofemoral bypass, subsequently developed intestinal gangrene as a result of a rare complication of mesenteric ischaemia.

## CASE REPORT

**Figure 1:**
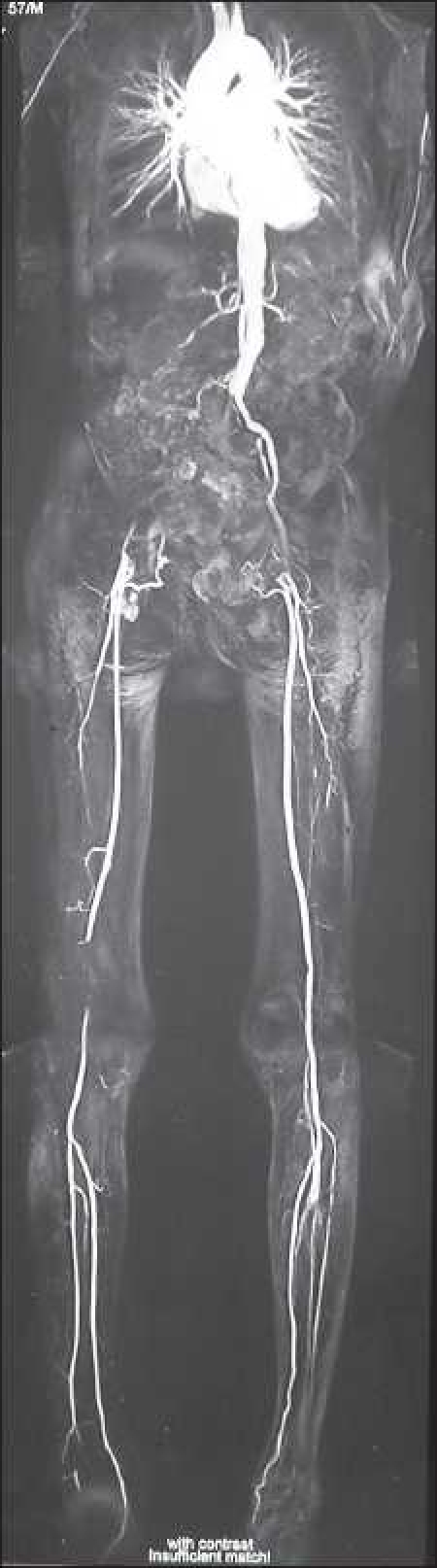
Magnetic resonance angiography: right common iliac, external iliac and popliteal artery blocked, left external iliac artery blocked

A 57-year-old male, weighing 50 kg, non-diabetic and chronic smoker with impending gangrene of the right foot was diagnosed to have obstruction of the right common iliac artery, external iliac artery, popliteal artery and left external iliac artery on magnetic resonance angiography [Figure 1]. Coronary angiography showed significant triple vessel disease (TVD). The patient was scheduled for urgent right-sided aorto-femoral bypass surgery as a limb saving procedure.

The patient was premedicated with intramuscular morphine 5 mg and promethazine 25 mg 1 h before surgery. In the operation theatre, electrocardiogram (ECG), pulse oximetry (SpO_2_) and invasive radial artery pressure monitoring were started. Anaesthetic induction included intravenous fentanyl 200 *µ*g, midazolam 2 mg, thiopentone 100 mg and trachea intubated with rocuronium bromide 50 mg. Anaesthesia was maintained with fentanyl, midazolam, vecuronium bromide, isoflurane 0.5–1% in the air–oxygen mixture and positive pressure ventilation to maintain normocapnia. Additional monitoring included end-tidal carbon dioxide, central venous pressure, temperature, urine output, electrolytes, arterial blood gases, blood glucose and transesophageal echocardiography (TEE).

Right aorto-femoral bypass was done with the aorta cross clamped. Infra-renal aortic cross clamp application caused a rise in systolic arterial pressure by 30 mmHg, ST segment depression in inferior leads and new regional wall motion abnormalities (RWMAs) in the anteroseptal wall on TEE in spite of nitroglycerine infusion and deepening with isoflurane. The infra-renal clamp was released. A partial aortic side clamp was applied at the bifurcation of the aorta which did not produce any ST segment changes or RWMAs. On the completion of aorto-femoral bypass, staged declamping was preceded by discontinuation of vasodilators. Sodium bicarbonate and intravenous fluids were infused to counter the resultant hypovolaemia and acidosis. After surgery, the patient was shifted to the intensive care unit (ICU), extubated 4 h later and observed for 72 h for any evidence of myocardial ischaemia. The preparation for an emergency coronary artery bypass graft (CABG) surgery was kept ready. The patient was discharged on the seventh postoperative day and planned for an elective CABG at a later date.

The patient was re-admitted 3 weeks later with complaints of abdominal pain and constipation of 3–4 days, abdominal distention and vomiting for 2–3 days. He had sunken eyeballs, a heart rate of 110 beats/minute, weak thready pulse and an arterial pressure of 76/48 mmHg. Chest auscultation revealed bilateral ronchi and basal crepitations. The abdomen was distended and tender. He was given intravenous fluids. Dopamine infusion 5 *µ*g/kg/min and correction of electrolyte imbalance was done. Blood investigations revealed haemoglobin of 12 g%, a total leukocyte count of 16,700 cells/mm^3^ with predominant neutrophilia of 86%, blood urea of 70 mg/dl and serum creatinine of 1.4 mg/dl. Abdominal erect radiograph showed dilated small bowel loops with no air under the diaphragm. Ultrasound of the abdomen showed dilated small bowel loops with the presence of free fluid. Computed tomography (CT) of the abdomen was not done as the patient had clear signs of peritonitis and was taken up for urgent laparotomy. Laparotomy revealed the gangrenous segment of the small bowel which was resected and end-to-end anastomosis of the distal ileum done under balanced general anaesthesia with precautions to maintain a favourable myocardial oxygen supply–demand balance. The patient was extubated and shifted to ICU. Gangrene of the right lower limb however progressed and right-sided-below-knee amputation was done under general anaesthesia 2 weeks later.

## DISCUSSION

Patients with coexisting peripheral vascular disease and significant coronary artery disease (CAD) requiring revascularization present a management dilemma. The operative mortality for peripheral vascular surgery is less if myocardial revascularization is done first (1–2%) as compared to patients without preliminary CABG (2–4%). The combined mortality for both CABG and vascular surgery is however higher (5–6%).[1,2] Need for peripheral vascular surgery versus CABG has to be individualized. Critical limb ischaemia in the present case necessitated an urgent peripheral vascular surgery first. The patient was monitored for myocardial ischaemia with ECG and TEE. Vascular surgeries involving aortic clamping and declamping often lead to haemodynamic disturbances and exacerbate underlying coronary ischaemia. Their impact can be minimized by using vasodilators and ensuring adequate anaesthetic depth during the period of clamping. In our patient, along with these, a partial aortic side clamp was applied at a lower level (at aortic bifurcation). The recent trend of endovascular stenting was not technically possible in our patient because of complete occlusion of the external iliac artery.[3]

Extensive atherosclerotic disease should also increase vigilance towards other organ systems which may be hypoperfused. The development of symptomatic intestinal ischaemia after abdominal surgery in patients with atherosclerotic disease has been described.[4] The presumed mechanism is the division of vital collaterals during the surgical procedure. This sequence of events has been most frequently recognized after abdominal vascular surgery (e.g. aortic aneurysm or renal artery repair). Also there can be worsening of ischaemia in patients who have a pre-existing atherosclerosis of the mesenteric arteries. A high index of suspicion is therefore required in the presence of vague nonspecific clinical presentation.

The risk factors for mesenteric artery ischaemia include age more than 65 years, cardiac arrhythmias, atherosclerosis, low cardiac output state, cardiac valvular disease and intra-abdominal malignancy.[5,6] The clinical presentation includes acute onset of severe abdominal pain if embolus is the cause. A gradual onset of pain which is visceral, poorly localized and classically out of proportion compared to findings on examination is however more common in the overall spectrum of mesenteric ischaemia. Nausea and vomiting are often present. Occult blood in rectum is seen in more than half of the cases.[6] Currently, there is no serum marker for establishing the diagnosis. The assay of intestinal fatty acid -binding protein (I-FABP), a marker of intestinal infarction, is not helpful in early diagnosis of the disease process.[7] D-dimer is a potential marker for early diagnosis. One study has reported 100% sensitivity but only 38% specificity for superior mesenteric artery thromboembolism.[8] Leukocytosis, increased lactate levels, or metabolic acidosis, are either not adequately specific or appear late in the disease process. Plain x-ray films of the abdomen are helpful in excluding other identifiable causes of abdominal pain. Duplex ultrasonograhy of mesenteric vessels is very sensitive(92–100%) in identifying proximal stenosis of vessels. Selective catheter angiography has been the gold standard for diagnosis of acute mesenteric ischaemia with a sensitivity of 90–100% and specificity of 100%.[9]

Recently CT of the abdomen and CT angiography are beginning to challenge traditional angiography as a diagnostic test for acute mesenteric ischaemia. A specificity of 94% and sensitivity of 96% can be achieved.[10] These tests should be performed early to prevent progression of mesenteric ischaemia to infarction. Magnetic resonance imaging does not add any additional diagnostic power over CT, but is advantageous where intravenous contrast is contraindicated.

In conclusion, the anaesthetic plan for patients with peripheral vascular disease should take into account the possibility of extensive atherosclerotic involvement of the vascular system of other body organs (coronary, cerebral, renal and mesenteric vessels). Cardioprotective strategies, maintenance of a favourable myocardial oxygen supply-demand and perioperative cardiac monitoring for myocardial ischaemia are crucial for a successful anaesthetic management. Mesenteric artery ischaemia can be one of the late presentations in the postoperative period in patients undergoing surgery for peripheral vascular disease.
